# Single-molecule localization microscopy and tracking with red-shifted states of conventional BODIPY conjugates in living cells

**DOI:** 10.1038/s41467-019-11384-6

**Published:** 2019-07-30

**Authors:** Santosh Adhikari, Joe Moscatelli, Elizabeth M. Smith, Chiranjib Banerjee, Elias M. Puchner

**Affiliations:** 1 School of Physics and Astronomy University of Minnesota, Twin Cities Physics and Nanotechnology (PAN), 115 Union Street SE, Minneapolis, MN 55455 USA; 2 Present Address: Middlebury College, 14 Old Chapel Road, Middlebury, VT 05753 USA

**Keywords:** Biological fluorescence, Single-molecule biophysics, Super-resolution microscopy, Fluorescence imaging

## Abstract

Single-molecule localization microscopy (SMLM) is a rapidly evolving technique to resolve subcellular structures and single-molecule dynamics at the nanoscale. Here, we employ conventional BODIPY conjugates for live-cell SMLM via their previously reported red-shifted ground-state dimers (D_II_), which transiently form through bi-molecular encounters and emit bright single-molecule fluorescence. We employ the versatility of D_II_-state SMLM to resolve the nanoscopic spatial regulation and dynamics of single fatty acid analogs (FAas) and lipid droplets (LDs) in living yeast and mammalian cells with two colors. In fed cells, FAas localize to the endoplasmic reticulum and LDs of ~125 nm diameter. Upon fasting, however, FAas form dense, non-LD clusters of ~100 nm diameter at the plasma membrane and transition from free diffusion to confined immobilization. Our reported SMLM capability of conventional BODIPY conjugates is further demonstrated by imaging lysosomes in mammalian cells and enables simple and versatile live-cell imaging of sub-cellular structures at the nanoscale.

## Abstract (web summary)

Single-molecule localization microscopy (SMLM) requires the use of fluorophores with specific sets of properties. Here the authors employ conventional BODIPY dyes as SMLM fluorophores by making use of rarely reported red-shifted ground state BODIPY dimers to image fatty acids, lipid droplets and lysosomes at single-molecule resolution.

## Introduction

Fluorescence microscopy methods allow for the specific investigation of proteins and subcellular structures in living cells. In conventional fluorescence microscopy, high-resolution information is lost due to the optical diffraction limit. Single-molecule localization microscopy (SMLM) techniques such as photo-activated localization microscopy and stochastic optical reconstruction microscopy (STORM) overcome the optical diffraction limit by building super-resolution images from precise localizations of single fluorophores across thousands of data-acquisition frames^1,2^. A requirement for fluorophores compatible with SMLM is, therefore, the ability to separate the fluorescence of single emitters in time in order to avoid spatial overlap of their point spread functions (PSF). The sparse subset of activated fluorophores in each frame is then precisely localized by fitting to obtain a high-resolution image of immobile structures or to track single molecules in living cells. The density of active fluorophores in fixed cells is commonly controlled by the photo-switching of fluorescent proteins^1^ or dyes^2,3^, the spontaneously induced blinking of fluorescent dyes by chemicals^4^, and the transient binding and unbinding of fluorophores^5^ or fluorogens^6^. Similar approaches with fluorescent proteins^7–9^, organic fluorophores^10–12^, and fluorogens^13^ have been developed in living cells. Here, we report a novel strategy and class of fluorophores for live-cell SMLM: the imaging of sparse, red-shifted ground state dimers, which transiently arise from bimolecular encounters of conventional boron di-pyromethane dyes (herein referred to as D_II_-BODIPY).

In ensemble spectroscopy experiments, BODIPY dyes have been shown to form ground state dimers with red-shifted absorption and emission through bimolecular encounters when their electronic orbitals are closer than 3.8 Å^14–16^. Further, it was established that the small fraction of these ground state dimers depends on the concentration of the monomers^14^. When two BODIPY dyes were chemically linked together in close proximity and in the right orientation through a molecular backbone, they exhibited exclusively the red-shifted excitation and emission of D_II_ states^17,18^. Here, we find that, compared to their monomeric states, single D_II_-BODIPY states can be specifically excited and detected with red-shifted wavelengths. The resulting single-molecule fluorescence exhibits a high photon budget over multiple frames, enabling high-resolution localizations in live-cell SMLM applications. Furthermore, the concentration of the monomeric BODIPY can be used to tune the density of single-molecule localizations to the typical range of SMLM experiments.

Due to their favorable spectroscopic properties^19^, hundreds of BODIPY conjugates labeling different cellular compartments and biomolecules have been developed for conventional fluorescence microscopy applications^20–23^. While photo-switchable versions of BODIPY probes have recently been reported^10,24,25^, the general applicability of all existing conventional BODIPY conjugates for SMLM opens up a simple and versatile method to obtain quantifiable super-resolution images as well as single-molecule dynamics in living cells using multiple colors.

In this work, we develop the multi-color SMLM capability of various conventional BODIPY conjugates to quantify the nanoscopic spatial distribution and the dynamics of single fatty acid analogs and lipid droplet (LD) probes in living yeast cells with ~30 nm resolution. The cellular uptake of fatty acid analogs and their subsequent use for membrane expansion or storage in LDs is a fundamental and well-known process across many species and cell types^26,27^. Likewise, the breakdown of LDs during lipolysis and lipophagy and the release of fatty acids for energy production have been extensively studied^28,29^ in the past. Both processes are involved in metabolic diseases and in cell survival during starvation^30^. However, only recently have LDs been recognized as actively regulated organelles^31^. Consequently, very little is known about how the metabolic state of cells regulates the dynamics and the nanoscopic spatial distribution of fatty acids and neutral lipids. Here, we track fatty acid analogs and resolve LDs with two BODIPY conjugates of different color in both fed and fasted yeast cells. Our results show dramatic changes in the diffusion and nanoscopic localization of fatty acid analogs and LDs and suggest a spatial protection mechanism against lipotoxicity. Furthermore, we demonstrate that these BODIPY probes are equally applicable for SMLM in living mammalian cells by resolving and tracking single fatty acid analogs, as well as lysosomes, using a BODIPY-Lysotracker. This general approach of localizing single D_II_-BODIPY states opens up a myriad of possibilities for simple SMLM experiments with the existing variety of conventional BODIPY probes.

## Results

### Live-cell SMLM with single red-shifted D_II_-BODIPY states

**Fig. 1:**
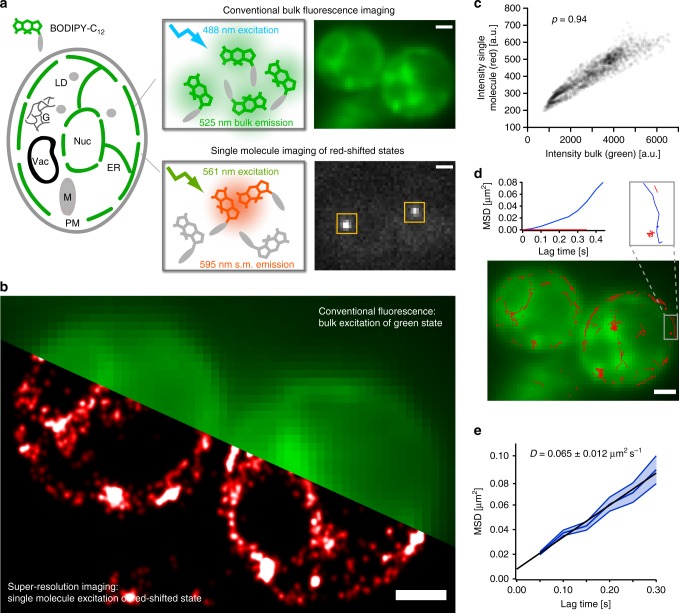
SMLM and tracking with transient red-shifted D_II_-states of conventional BODIPY conjugates. **a** BODIPY fluorophores exist as various conjugates that label specific compartments within cells. BODIPY-C_12_ is a fatty acid analog that localizes to the ER of living yeast cells when excited at 488 nm in the conventional fluorescence microscopy mode (green, upper). When excited at 561 nm, bright and red-shifted single-molecule fluorescence signals appear and disappear throughout the cell (white, lower). **b** The bright single-molecule fluorescence can be used for SMLM to obtain a ~tenfold higher resolution image of the fatty acid distribution within the cell (red). **c** The pixel intensities of the conventional fluoresce image of BODIPY-C_12_ excited at 488 nm correlate well with the single-molecule signal excited at 561 nm and averaged over 800 frames (Pearson’s *ρ* = 0.94, number of cells = 2, number of data points = 2128), confirming the common origin of the two signals. **d** The detected single-molecule signal of D_II_-BODIPY states lasts long enough to track fatty acid analogs (red traces). Different mobile (blue) and immobile (red) species can be discriminated. **e** The average mean square displacement vs. time of fatty acid analogs of 150 traces each lasting for at least 3 acquisition frames (0.15 s) results in an average diffusion coefficient of 0.065 ± 0.012 µm^2^ s^−1^ (error band: s.e.m., scale bars: 1 µm)

In order to gain high-resolution insights into the dynamics and localization of fatty acids, we developed and optimized the single-molecule imaging capability of D_II_-BODIPY states using BODIPY-C_12_, which is a fluorescently labeled fatty acid analog. BODIPY-C_12_ localizes to the endoplasmic reticulum (ER) and LDs and has been widely used to follow the uptake and distribution of fatty acid molecules with conventional fluorescence microscopy^26,28,32^. When living yeast cells (*S. cerevisiae*) were incubated with BODIPY-C_12_ for 30 min and excited with low intensities of 488 nm laser light, we observed the expected bulk fluorescence from the ER around the nucleus and plasma membrane (see Fig. 1a, upper). A few dense clusters indicated the incorporation of fatty acid analogs into LDs as later confirmed through co-localization with a LD marker. However, when cells were excited at 561 nm with a high-laser power typical for single-molecule imaging, bright and spatially well-separated spots intermittently turned “on” and “off” in a binary fashion (Fig. 1a, lower and Supplementary Movie 1). This observed signature is characteristic for single-molecule fluorescence and is presumably caused by transient D_II_-BODIPY states, which have been previously identified with ensemble fluorescence spectroscopy and shown to have an absorption maximum at 577 nm^15^ (see also “Discussion”). Importantly, the photon budget and appearance rate of single D_II_-BODIPY emitters are perfectly suitable for SMLM as they allow for the precise fitting and localization of their PSFs over tens of thousands of frames (Fig. 1b). The high degree of co-localization between the conventional and averaged single-molecule fluorescence signal (Pearson’s correlation *ρ* = 0.94, Fig. 1c and Supplementary Fig. 1a, b) further confirms that BODIPY causes the observed red-shifted single-molecule fluorescence. We note, however, that SMLM images look different compared to conventional epifluorescence images due to the increase in contrast (only molecules in focus are localized and no out of focus background is visible) and in resolution (each molecule is rendered with a ~tenfold smaller PSF). The increase in contrast is further amplified when imaging D_II_-states due to their quadratic dependence on the monomer concentration as explained and quantified below.

Since BODIPY probes are membrane permeable and compatible with live-cell imaging, the single-molecule signal of their D_II_-states can also be used for high-resolution single-molecule tracking^7^ (Fig. 1d). Interestingly, we found a wide variety in the diffusive behavior of BODIPY-C_12_ molecules, with some exhibiting free and normal diffusion along the ER, while others were immobilized (see zoom in Fig. 1d). The average diffusion coefficient of 0.065 ± 0.012 µm^2^ s^−1^ (Fig. 1e and Supplementary Fig.1c), calculated from the mean squared displacement (MSD) vs. time is within the range of other membrane localized molecules^7,33^. We interpret and later confirm that these immobile accumulations of fatty acid analogs localize to LDs. These results demonstrate that the discovered single-molecule fluorescence of red-shifted D_II_-BODIPY states is well suited for super-resolution imaging and single-molecule tracking in living cells.

### Optimized SMLM conditions for red-shifted D_II_-BODIPY states

**Fig. 2:**
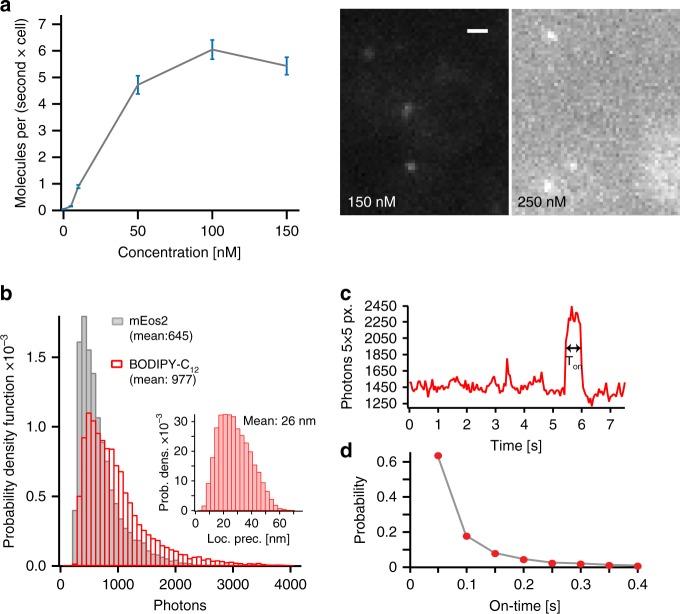
Photophysical characterization and optimization D_II_-BODIPY-C_12_ for SMLM. **a** The rate of single-molecule detections is dependent on the added concentration of BODIPY-C_12_ (left) (number of cells > 10, frames > 3500 for each concentration, error: standard deviation from 5 substacks). At concentrations above 150 nM the background fluorescence prevents single-molecule detection (right). **b** The photon counts detected from single red-shifted D_II_-BODIPY states per frame is superior to mEos2, a commonly used photo-switchable fluorescent protein (number of data points: mEos2 = 11,569, BODIPY-C_12_ = 25,673). **b** (inset) The theoretical localization precision calculated from the photon counts has a mean of 26 nm (*n* = 3754 points). **c** The single-molecule fluorescence intensity vs. time trace shows prolonged on-times, allowing for single-molecule tracking over several frames. **d** The on-time distribution follows an exponential decay with ~40% of molecules appearing in two or more frames for single-molecule tracking (right) (*n* = 6569 data points, 5 cells). Scale bar: 1 µm

An important parameter to consider when using BODIPY probes for SMLM is the rate of detecting red-shifted D_II_-states, which is expected to depend on the concentration of the BODIPY monomers. We, therefore, performed and analyzed SMLM experiments at various BODIPY-C_12_ concentrations and quantified the rate of single-molecule localizations (Fig. 2a). The localization rate increased with an increasing extracellular monomer concentration up to 150 nM where single emitters were still sparse enough to be individually localized. However, at a concentration above 150 nM, the increasing background fluorescence reduced the number of detectable molecules (Fig. 2a, right). Importantly, the background fluorescence of D_II_-BODIPY states in solution exhibited a quadratic dependence on the monomer concentration, consistent with their formation through bimolecular encounters (see Supplementary Fig. 2b). A second important parameter that directly affects the resolution of SMLM data is the number of photons detected from each fluorophore. We, therefore, measured the photon histogram of D_II_-BODIPY-C_12_ and mEos2, a widely used photo-convertible fluorescent protein for super-resolution microscopy^34^ (Fig. 2b). The mean photon yield of D_II_-BODIPY-C_12_ was higher compared with mEos2 and resulted in an average theoretical localization precision of 26 nm according to the formula of Thompson et al.^35^ (Fig. 2b, inset). The long tail of high-photon counts contained 10% of localizations with a precision better than 15 nm, which would allow for filtering of high-resolution localizations if desired. When optimizing the excitation laser power, we found an expected improvement of the theoretical resolution with increasing power (Supplementary Fig. 2a). The ability to precisely track single D_II_-BODIPY states further depends on the likelihood to detect a single emitter longer than one frame. When we analyzed the on-time distribution, we found ~40% of individual emitters to be detectable in two or more consecutive frames at 50 ms exposure time, allowing us to track a significant number of D_II_-BODIPY-C_12_ states (Fig. 2c, d).

### Versatile two-color SMLM and tracking with D_II_-BODIPY states

**Fig. 3:**
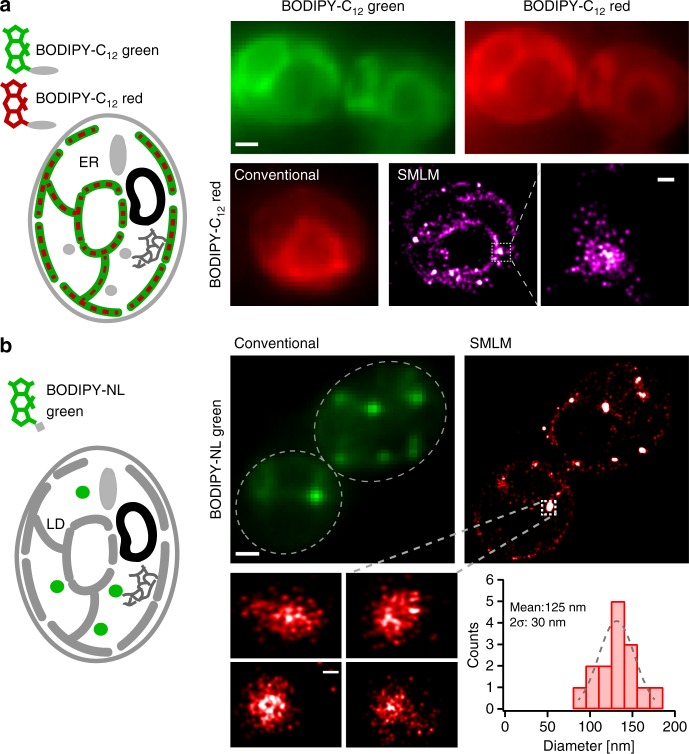
General applicability of versatile BODIPY conjugates for multi-color SMLM. **a** Conventional images of BODIPY-C_12_ green and BODIPY-C_12_ red show co-localization of both fatty acid analogs (top). Conventional fluorescence and super-resolution images of BODIPY-C_12_ red (bottom). **b** Conventional fluorescence and super-resolution images of LDs using BODIPY-NL. Zooms show super-resolution images of individual LDs that cannot be resolved with conventional fluorescence microscopy. The quantified diameters of different LDs have a mean of 125 nm. Scale bar: 1 µm, zoom: 100 nm

In order to expand the versatility of imaging D_II_-BODIPY with SMLM to multiple colors, we used BODIPY-C_12_ red, a red version of the BODIPY-fatty acid analog. Based on our previous findings of the red-shifted single-molecule fluorescence of D_II_-BODIPY states, we hypothesized that D_II_-BODIPY-C_12_ red would exhibit absorption and emission shifted to a far-red wavelength. When excited at the regular 561 nm wavelength, we observed a conventional fluorescence signal with a similar distribution compared to BODIPY-C_12_ around the ER of yeast cells (Fig. 3a, left). However, when excited at a far-red shifted wavelength of 640 nm and high laser power typical for single-molecule imaging, we again observed single-molecule fluorescence presumably caused by red-shifted ground state dimers as in the case of D_II_-BODIPY-C_12_. The high photon yield, on-time distribution, and appearance rate again allowed us to perform SMLM and to reconstruct super-resolution images in the far-red channel (Fig. 3a, lower right).

The large variety of existing BODIPY probes labeling specific compartments and biomolecules enables versatile opportunities for SMLM. We, therefore, tested BODIPY-NL, another green membrane permeable BODIPY version that has been widely used to selectively label LDs and to quantify the amount of neutral lipids in cells^36,37^. After the initial stage of LD biogenesis within the ER membrane, LDs split off into the cytoplasm of the cell where they store triglycerides and sterol esters surrounded by a phospholipid monolayer^31^. Due to their size below the optical diffraction limit in yeast (60–300 nm)^38,39^, biogenic and matured LDs cannot be resolved with conventional fluorescence microscopy and thus present an ideal application for super-resolution imaging. Upon excitation with the conventional 488 nm wavelength, BODIPY-NL exhibited the expected punctate distribution consistent with the specific labeling of LDs (Fig. 3b). When excited with a high power of a 561 nm laser, however, we again observed single-molecule fluorescence in the red-shifted channel and reconstructed super-resolution images of LDs (Fig. 3b, right) with a calculated average localization precision of 28 nm (Supplementary Fig. 3b). The size distribution of LDs (Fig. 3b, lower) exhibited a peak at 125 nm, which agrees with previous EM studies^38^ and highlights the potential to quantify and resolve subcellular structures with ~tenfold higher resolution in living cells compared to conventional fluorescence images. To assess whether SMLM data of D_II_-BODIPY states can be quantified, we plotted the number of localizations from individual LDs of different size against their monomer fluorescence intensity (Supplementary Fig. 3d). The resulting correlation coefficient of *ρ* = 0.9 is similar to a study using mEos2-tagged endosomes (*ρ* = 0.91)^40^ and indicates that quantification is indeed feasible. Due to its localization inside LDs, the diffusion of D_II_-BODIPY-NL was predominantly confined (Supplementary Fig. 3c) in contrast to the free diffusion observed for the fatty acid analogs. These results demonstrate that conventional BODIPY conjugates with different colors and functional groups present a versatile approach for multi-color super-resolution imaging and single-molecule tracking in living cells.

### Nanoscale behavior of FAa depends on metabolic state of cell

Previous conventional fluorescence microscopy studies revealed that the uptake and distribution of fatty acids are actively regulated by the metabolic state of cells^28^, which is critical for cell survival under starvation and may be disrupted in disease states^41^. While the high resolution of electron microscopy in fixed cells also contributed to our understanding of lipid metabolism, these traditional techniques cannot provide high-resolution information about specific biomolecules such as fatty acid analogs or neutral lipids in living cells. We, therefore, sought to investigate with our developed SMLM capability of D_II_-BODIPY conjugates how the nanoscopic spatial distribution and mobility of fatty acids is regulated by the metabolic state of live yeast cells.

**Fig. 4:**
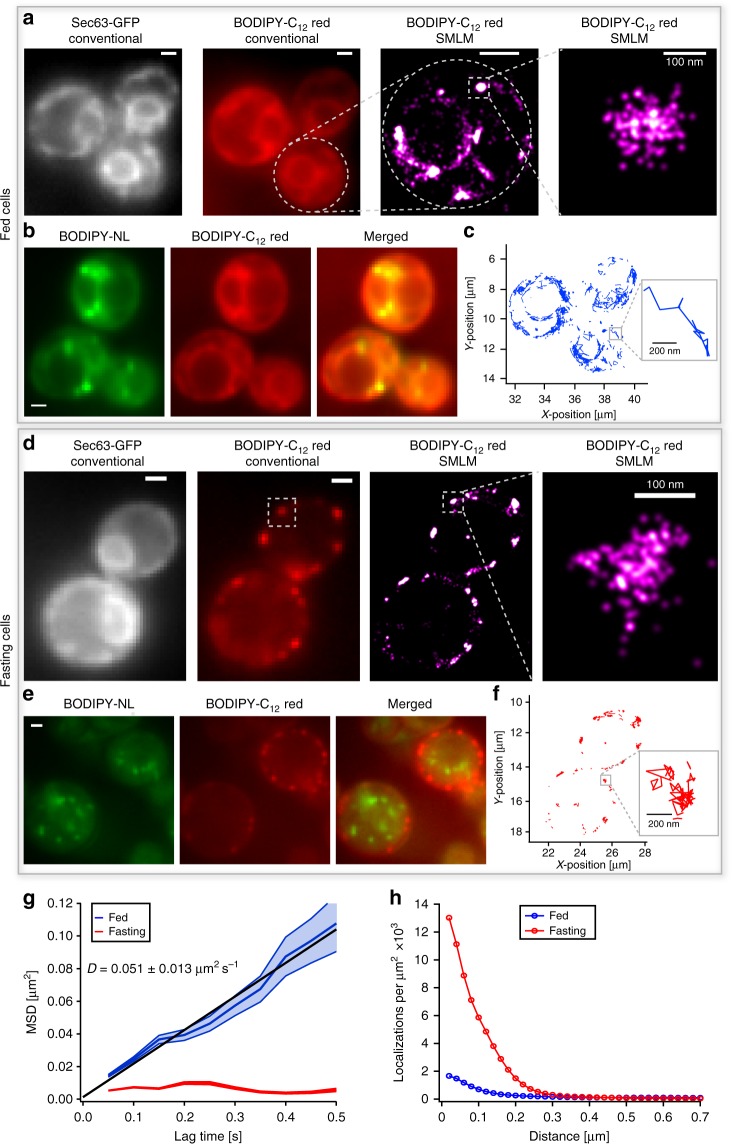
Differential nanoscale localization and dynamics of fatty acid analogs depend on metabolic state of cells. **a** Conventional fluorescence images of BODIPY-C_12_ red exhibit co-localization with the ER protein Sec63-2 × GFP under fed condition. The SMLM image of BODIPY-C_12_ red shows the ER membrane in high resolution and distinct spots on the ER below the optical diffraction limit. **b** The co-localization of BODIPY-C_12_ red with BODIPY-NL shows that denser spots of BODIPY-C_12_ co-localize with LDs. **c** The single-molecule tracking of BODIPY-C_12_ red reveals predominantly free diffusion along the ER membrane. **d** Under fasted conditions, added BODIPY-C_12_ red forms distinct puncta along the cell periphery that do not co-localize with Sec63-2 × GFP at the nuclear portion of the ER. The super-resolution images resolve puncta below the diffraction limit. **e** The distinct BODIPY-C_12_ red puncta do not co-localize with LDs (BODIPY-NL) in fasting cells. **f** The single-molecule tracking of BODIPY-C_12_ red exhibits mostly confined immobilization in fasting cells. **g** The MSD vs. time of BODIPY-C_12_ red molecules shows free diffusion (*D* = 0.051 ± 0.013 μm^2^ s^−1^) under fed conditions (blue) and confined immobilization under fasted conditions (red) (Fed: 321 traces; Fasted: 151 traces; error: standard deviation from 6 movies each with > 3 cells). **h** The radial distribution function shows high-density clusters of BODIPY-C_12_ red localizations upon fasting (red) and a much smaller fraction of clustered localizations in fed cells (blue) (Fed: 4219 localizations, 2 cells; Fasted: 4933 localizations, 2 cells). Scale bar: 1 µm unless specified

To confirm uptake of the labeled fatty acids and their subsequent incorporation into the ER, we first imaged Sec63-2 × GFP, a protein specifically found in the membrane of the ER^42^ and then added BODIPY-C_12_ red for co-localization experiments. After 30 min of incubation, the conventional fluorescence images of BODIPY-C_12_ red, as well as the super-resolution images of its D_II_-states, showed the expected co-localization with Sec63-2 × GFP (Fig. 4a). The increase in resolution of SMLM images revealed much finer details compared to conventional fluorescence images: a population of fatty acid analogs that is evenly distributed along the ER and another that is accumulated in dense clusters. We hypothesized that these clusters present the incorporation of the labeled fatty acids into newly forming or pre-existing LDs in the ER membrane (Fig. 4a, right). To verify the origin of these dense fatty acid accumulations, we added the neutral lipid probe BODIPY-NL to the same cells. The co-localization with neutral lipid puncta indicated that these dense clusters of fatty acid analogs are indeed localized to LDs (Fig. 4b, Supplementary Fig. 4a, *ρ* = 0.8). Furthermore, single molecule tracking of D_II_-BODIPY-C_12_ revealed that fatty acid analogs are immobile in LDs, but show free diffusion along the ER membrane (Fig. 4c). The size of individual LDs (Fig. 4a, right) corresponds with EM studies^38^, and demonstrates the promise of our approach to resolve and study these emerging structures in living cells.

In order to uncover how the metabolic state of cells affects the distribution and dynamics of fatty acid analogs and LDs, we fasted the same yeast culture by growing them in the stationary phase for ~30 h (no change in optical density of cells). Sec63-2 × GFP still showed its regular ER localization (Fig. 4d, Supplementary Fig. 4b). However, BODIPY-C_12_ red added again 30 min before imaging exhibited a remarkably different localization pattern with distinct puncta at or near the plasma membrane and no detectable signal from the ER portion around the nucleus (Fig. 4d and Supplementary Fig. 4b, right). These puncta were immobile and their super-resolution images revealed a diameter around 100 nm (Fig. 4d, right and Fig. 4h), which is similar to the observed size of LDs in Fig. 3b. To test if the fatty acid analogs are still accumulated in LDs as in the fed state, we again added BODIPY-NL to stain for neutral lipids. Remarkably, we observed no co-localization of fatty acid analogs with LDs (Fig. 4e, Supplementary Fig. 4c, *ρ* = 0.3). The detected LDs were either moving randomly inside the vacuole or were docked to the vacuolar membrane as previously observed^43^ (Supplementary Movie 2). Since starving cells undergo lipophagy, the observed shut-down of fatty acid transport to the ER and their aggregation into puncta may therefore protect cells from lipotoxicity caused by cytoplasmic fatty acids (see also Discussion).

We also utilized our ability to track fatty acid analogs under fed (Fig. 4c) and fasted (Fig. 4f) conditions and observed strong differences in their mobility. Under fed conditions, fatty acid analogs exhibited a mean diffusion coefficient of *D* = 0.051 ± 0.013 µm^2^ s^−1^ (mean ± SD) (Fig. 4g), which is within the range of membrane-localized molecules^7,33^ and consistent with their ER localization. However, under starved conditions we found a dramatically reduced mobility of fatty acids with no component of free diffusion but only confined motion (Fig. 4g). This confinement to ~100 nm is consistent with the measured size of puncta (Fig. 4h) and may contribute to reducing lipotoxicity by preventing free diffusion of fatty acid analogs throughout the cytoplasm of the cell.

**Fig. 5:**
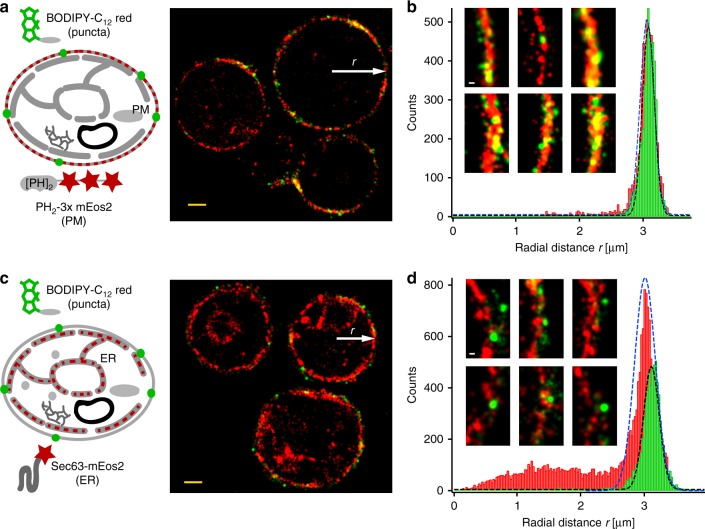
Two color SMLM reveals co-localization of fatty acid analog puncta with the plasma membrane in fasting cells. **a** Sequential SMLM imaging of D_II_-BODIPY-C_12_ red puncta and a double PH domain of PLCdelta(1) fused to 3x mEos2 shows co-localization of fatty acid puncta with the plasma membrane in fasting cells. **b** The radial distance histogram of BODIPY-C_12_ red and PH_2_-3xmEos2 localizations from five cells (scaled to the same radius) confirms the co-localization with no detectable separation outside the localization error. The insets show magnifications of rendered SMLM images and overlap of BODIPY-C_12_ red puncta with the plasma membrane. **c** Sequential SMLM imaging of D_II_-BODIPY-C_12_ red puncta and Sec63-mEos2 shows no co-localization of fatty acid puncta with the cortical ER close to the plasma membrane. **d** The radial distance histogram of BODIPY-C_12_ red and Sec63-mEos2 localizations from five cells (scaled to the same radius) reveals a mean separation of fatty acid puncta from the ER of ~100 nm. The insets show magnifications of rendered SMLM images and the separation of BODIPY-C_12_ red puncta from the ER. Scalebar: 1 µm; insets 100 nm

To discriminate whether the puncta of fatty acid analogs in fasted cells are localized to the plasma membrane or to the nearby ER, we performed SMLM co-localization experiments. We first imaged the double PH-domain of PLCdelta(1) fused to mEos2 as a plasma membrane marker at high 405 nm activation power to achieve high mEos2 localization densities. Next, we imaged BODIPY-C_12_ at low concentrations (50 nM) in the absence of 405 nm light to minimize potential crosstalk from mEos2 (Fig. 5a and Supplementary Information). The SMLM images and radial density distribution (Fig. 5b), as well as the cross-correlation (Supplementary Fig. 5) show that BODIPY-C_12_ puncta in fasted cells indeed co-localize with the plasma membrane with no measurable separation beyond our localization precision. In contrast, when we performed similar experiments with Sec63-mEos2 as an ER marker (Fig. 5c), we found a mean separation of plasma-membrane portion of the ER from BODIPY-C_12_ puncta of ~100 nm (Fig. 5d). These high-resolution measurements clearly indicate that BODIPY-C_12_ puncta in fasted cells do not co-localize with the ER but with the plasma membrane.

### Extending SMLM of D_II_-BODIPY states to live mammalian cells

**Fig. 6:**
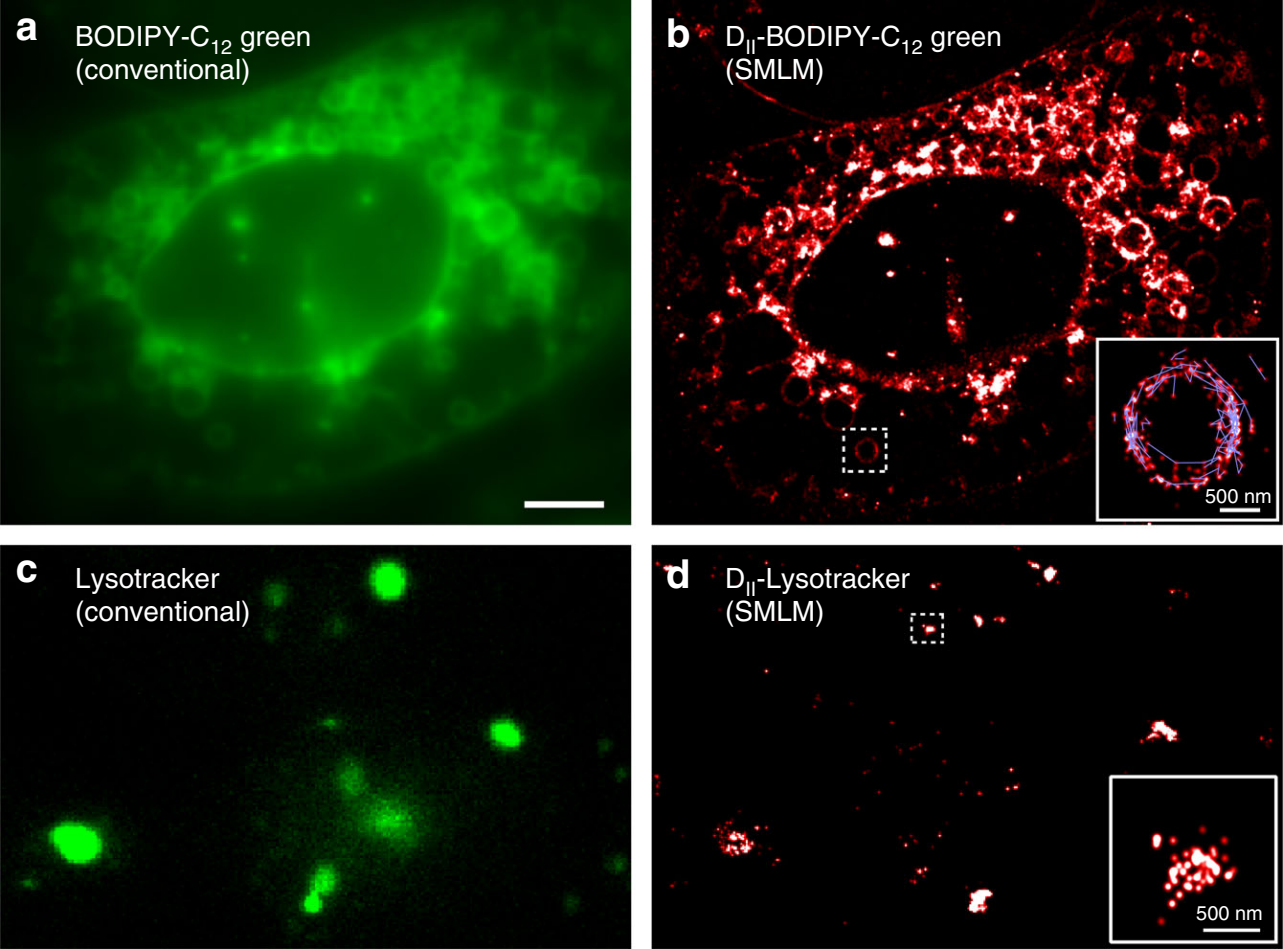
SMLM and tracking of fatty acid analogs and LysoTracker Green using D_II_-states in living mammalian cells. **a** Conventional fluorescence image of BODIPY-C_12_ in U2OS cells. LDs of various sizes become visible and are generally bigger compared to yeast. **b** Super-resolution microscopy image of D_II_ states of BODIPY-C_12_. Zoom: Superposition of single-molecule localizations (red) and traces (blue) reveal the motion of fatty acid analogs along the periphery of LDs. **c** Conventional fluorescence image of LysoTracker Green with 488 nm excitation. **d** SMLM images of D_II_ states of LTG with 561 nm excitation. Zoom**:** SMLM image of an immobile lysosome. Scale bar: 5 µm; insets: 500 nm

In order to validate the general applicability of D_II_-BODIPY states for SMLM in different cell types, we studied the distribution of fatty acid analogs in living mammalian U2OS cells using BODIPY-C_12_. The conventional fluorescence microscopy images of BODIPY-C_12_ (excitation wavelength 488 nm) showed the expected outline of large and resolvable lipid droplets^27^ (Fig. 6a, left). We also observed fainter staining of the plasma and the nuclear membrane, which has recently been thought of as a major active site for LD biogenesis^44^. When excited at high power of 561 nm laser light, we detected bright single-molecule fluorescence similar to yeast and suitable for generating super-resolution images. The lower background and higher resolution compared to the conventional fluorescence images revealed much finer details such as thin protrusions of the ER and LDs with a size below the optical diffraction limit (Fig. 6b). The SMLM capability of D_II_-BODIPY states again allowed us to track fatty acid analogs, which exhibited diffusion along the circular surface of LDs and other membrane structures (Fig. 6b, zoom). The average diffusion coefficient of fatty acid analogs was calculated from the MSD vs. time plot to be 0.083 ± 0.015 µm^2^ s^−1^, which is similar to yeast and within the published range for membrane localized molecules (Supplementary Fig. 6a).

To further demonstrate the universal applicability of BODIPY D_II_-states for SMLM, we used another BODIPY analog, LysoTracker Green (LTG) and generated super resolution images of lysosomes in U2OS cells. The conventional fluorescence image of LTG (488 nm excitation, Fig. 6c), shows spherical lysosomes of various sizes. When LTG was excited with high 561 nm laser power we again detected single molecule fluorescence and were able to reconstruct super resolution images of lysosomes (Fig. 6d). While several lysosomes appear as elongated structures caused by their movement during the data acquisition time of several minutes, we are able to resolve some diffraction limited and immobile lysosomes by restricting the frame range used for analysis (Fig. 6d, zoom).

These results demonstrate that the SMLM capability of conventional BODIPYs is applicable across different cell types and organisms and open up a myriad of opportunities for future studies using the variety of existing BODIPY conjugates.

## Discussion

BODIPYs are one of the most widely used dyes for conventional fluorescence microscopy to image various cellular compartments, membranes, and biomolecules. Their commercial availability in multiple colors and as various conjugates with different functional groups make them a versatile and user-friendly class of fluorophores compatible with live-cell imaging. In previous ensemble spectroscopy experiments it has been shown that BODIPY dyes form ground state dimers with red-shifted absorption and emission through coupling of their electronic states^14–16^. These D_II_-BODIPY states have also been specifically synthesized and characterized by coupling two BODIPY dyes in close proximity and proper orientation to a scaffold molecule^17,18^. Here, we discovered that single D_II_-BODIPY states emit bright single-molecule fluorescence comparable to other commonly used fluorophores and can be used for multi-color super-resolution microscopy and single-molecule tracking. Our characterization and optimization provide direct and versatile applicability. However, future photophysical and photochemical studies will be necessary to fully understand the photophysics of BODIPY probes and to further optimize this system, e.g., for even higher photon budgets and longer on-states for single-molecule tracking. For instance, a few conventional bulk fluorescence experiments showed that some BODIPY probes can be photoconverted by blue (488 nm) light in certain cellular environments to a species that is spectrally similar to D_II_ states. However, since this species was kinetically stable for more than 15 min^45,46^, it cannot be explained by transient D_II_-state formation. While we did not observe any blue light-induced fluorescence of our BODIPY probes in the red excitation and emission channel, this effect would not interfere with our reported SMLM capability of D_II_ states and could even be exploited to externally control the localization density.

Due to the nature of single emitter formation through transient bimolecular encounters, no high-energy wavelengths for photoswitching are necessary to activate D_II_-BODIPY states. This is a notable improvement when comparing our technique to photo-switchable versions of BODIPY^10,24^ or other organic dyes compatible with live cell SMLM; it prevents phototoxicity^47^ and the potential need for chemical modifications. Many of the developed BODIPY probes possess superb specificity for cellular compartments, which is further amplified by the bimolecular encounters leading to the detected red-shifted D_II_-states. Consequently, the strikingly simple sample preparation just requires adding the BODIPY conjugates at ~100 nM to the cell culture without any washing or exchange with imaging buffers. Compared to traditional SMLM labeling strategies, another advantage of our system is the almost infinite pool of BODIPY monomers, which is unaffected by the excitation wavelength of the dimers. BODIPY monomers thus provide a lasting source of dimer formations for single-molecule localizations in long-term measurements of single cells. Our quantified lifetime of *τ* = 2333 ± 25 s (fit coefficient value ± one standard deviation, Supplementary Fig. 6b) exceeds by far the lifetime of recently introduced SiR-based HIDE probes for live cell SMLM (*τ* = 414 ± 27 s, mean ± s.e.m.)^48^. However, fixed-cell measurements under oxygen depletion resulted in longer estimated life times of 9000 s for a novel fluorogen system^49^ and even more for Alexa dyes in an optimized imaging buffer^50^.

The ability to excite and image the monomeric pool of BODIPY in a spectrally distinct conventional fluorescence channel allows for the immediate recognition of a cell or organelle of interest without the need to collect and analyze long sequences of SMLM data. Of special interest for such correlative super-resolution and conventional microscopy studies is the exploitation of BODIPYs anti-Stokes shift, which results in a simultaneously detectable signal of the BODIPY monomers in their conventional fluorescence channel when excited with the red-shifted laser wavelength at the same intensity used for imaging their D_II_-states (see also Supplementary Fig. 1b). Our reported SMLM capability of conventional BODIPY conjugates opens up new avenues for the nanoscale imaging and quantification of numerous cellular structures in living cells.

In this work, we employed our developed capability of imaging conventional BODIPY conjugates with SMLM to study the spatial distribution and dynamics of neutral lipids and fatty acid analogs with unprecedented resolutions of ~30 nm inside living cells. Cells take up fatty acids and control their over-accumulation to avoid lipotoxicity^51,52^ through three processes: (a) esterification and conversion into neutral lipids (triacylglycerol, sterol esters) in the ER membrane for membrane expansion and storage in LDs^26^, (b) import and oxidation in mitochondria (mammalian cells) or peroxisomes (yeast)^53,54^ for ATP production, and (c) export by ATPases. Hence, under fed conditions when energy is not limited, externally added fatty acids are predominantly localized to the ER membrane and get to a smaller extent incorporated in LDs for storrage^55,56^. While BODIPY fatty acid analogs have been shown to perturb the phase separation of fatty acids in some biological contexts, their regular uptake, esterification, and incorporation into LDs have been demonstrated in yeast^26^ and mammalian cells^27^. During our incubation times of 30 min, no significant conversion of BODIPY fatty acid analogs has been reported, which could potentially lead to a mixture of BODIPY analogs^27^. Using our SMLM capability of fatty acid analogs along with the co-localization of Sec63 and neutral lipids, we demonstrated that fatty acids analogs indeed localized to the ER. A few denser regions co-localized with neural lipids and indicated their incorporation into LDs. Quantification of the size of these clusters revealed a mean diameter of 125 nm, which cannot be resolved with conventional fluorescence microscopy. The quantified single-molecule tracking of individual fatty acid analogs showed two different populations: (1) freely diffusing fatty acids along the membrane of the ER with a diffusion coefficient comparable to other membrane localized molecules and (2) a fraction of immobile fatty acid analogs that showed confined motion and localized to LDs.

When yeast cells are fasting, LDs accumulate around and within the vacuole, where fatty acids derived from neutral lipids get released in the process of lipophagy^29,43,57^. The released fatty acids are utilized for beta-oxidation in mitochondria (mammalian cells) or peroxisomes (yeast)^53,54^ to meet the energy needs of the cell. Under fasted conditions, our results showed that LDs predominantly localized to the vacuole and exhibited fast diffusion (Supplementary Movie 2) consistent with the current model of lipophagy^43^. More strikingly, fatty acid analogs that were added to fasted cells did not localize to LDs or to the ER membrane as in the case of fed cells, but formed clusters of ~100 nm diameter in proximity to the plasma membrane of the cells. This co-localization was further confirmed by two color SMLM, which revealed a mean distance of these puncta from the ER of ~100 nm and no detectable separation from the plasma membrane. In strong contrast to the freely diffusing species along the ER in fed cells, single-molecule tracking in fasted cells showed mostly immobile fatty acid analogs that were confined to these clusters. Since starving cells are vulnerable to lipotoxicity caused by the release of fatty acids during lipophagy, externally adding fatty acids present a burden^51,52^. Our observed accumulation of fatty acid analogs in puncta at the plasma membrane and the absence of their incorporation into LDs, therefore, may serve the dual role of conserving energy and of providing a spatial protection mechanism against lipotoxicity. Likewise, the immobility of fatty acid analogs in these puncta prevents them from spreading their lipotoxic effects throughout the cell. These observations clearly indicate that cells tightly regulate fatty acid import and distribution based on their metabolic states and energy needs. Both, the small size and the diffusive behavior were directly accessible using SMLM and could not be resolved with conventional fluorescence microscopy. Our finding of the SMLM capability of conventional BODIPY conjugates therefore lays the foundation for future studies on the mechanisms of this pathway and provides new avenues for investigating versatile biological processes below the optical diffraction limit.

## Methods

### Sample and dye preparation

The parental yeast strain for all experiments was W303_ADE2 (MATa ura3 trp1 leu2 his3 can1 ADE2) derived from the yeast W303 MATa strain (MATa ura3 trp1 leu2 his3 ade2 can1, Horizon-Dharmacon, YSC1058). The ade2 mutation in the purchased strain was repaired by polymerase chain reaction (PCR)-mediated transformation. Wild-type ADE2 was amplified from genomic DNA, RB201 (W303 MATa, trp1, leu2, ura3, his3, can1R, and ADE2)^58^ with Phusion PCR (NEB) using the forward primer (ATGGATTCTAGAACAGTTGGTATATTGGGAGGGGGACAA) and the reverse primer (TTACTTGTTTTCTAGATAAGCTTCGTAACCGACAGTTTCTAACTT). The Sec63-2XGFP plasmid (plasmid: pJW595, parent vector: pSV606, promoter: pSte5, domain: Sec63-2XGFP) was a kind gift from Jessica Walter and Wendell Lim (University of California, San Francisco). The plasmid was generated (but not published) in the same way as in Puchner et al.^40^ using combinatorial cloning methods^59^. We linearized and transformed the yeast-integrating vector into our W303_ADE2 strain with the lithium acetate method. FAA4 was tagged with mEos2 in its native chromosomal context within the W303_ADE2, Sec63-2XGFP strain using homologous recombination of a PCR product. The PCR product was generated from the tagging cassette pFA6a-mEos2HIS3^40^ (Parent Vector: pFA6a-GFPHIS3^60^) with Phusion PCR (NEB) using the forward primer:

(CGGCTGTCAAGCCAGATGTGGAAAGAGTTTATAAAGAAAACACTggaggcagcAGTGCGATTAAGCCAGA) and reverse primer:

(GTGTTTATGAAGGGCAGGGGGGAAAGTAAAAAACTATGTCTTCCTgaattcgagctcgtttaaac). Likewise, the Sec63-mEos2 strain with endogenously tagged Sec63 was made using homologous recombination of a PCR product. The PCR product was generated from the tagging cassette pFA6a-mEos2HIS3 with Phusion PCR (NEB) using the forward primer:

(ATACTGATATCGATACGGATACAGAAGCTGAAGATGATGAATCACCAGAAGGATCCGGTGCTTCTAGTGC).

and reverse primer:

(AATATATACGTCTAAGAGCTAAAATGAAAAACTATACTAATCACTTATATtatcatcgatgaattcgagctcgt). The yeast strain expressing the double PH-domain of PLCdelta(1) fused to a triple repeat of mEos2 was previously published^40^, and for this study we replaced the weak pINO promoter with 511 bases of the stronger pSte5 promoter (W303 genome).

Yeast strains were incubated overnight with shaking (270 r.p.m.) at 30 °C in synthetic complete dextrose (SCD) medium. A morning dilution of 1:50 in SCD was performed and cells were grown for 4 h to exponential phase. The chambered borosilicate coverglasses (eight well, Lab-Tek II; Sigma-Aldrich) were incubated with 80 µl of 0.8 mg/ml sterile Concanavalin A (Con A) in deionized H_2_O for 30 min and subsequently washed three times with deionized H_2_O (Millipore). Yeast cells were incubated on the coverglass and allowed to settle for 30 min in SCD at a desired optical density (OD) of ~0.12. Under fasting conditions, cells were grown to stationary phase in SCD (OD saturation; ~30 h) and diluted with SC (without glucose) medium to the OD of ~0.12 prior to imaging.

Mammalian ATCC U2OS cells (Manassas, VA) that were a kind gift from Dr. Jochen Mueller (University of Minnesota), were maintained in DMEM with 10% fetal bovine serum and 1% pen/strep antibiotics. Before imaging, the medium was exchanged with live-cell imaging solution from ThermoFisher (A14291DJ) to reduce auto-fluorescence. BODIPY-C_12_ or LysoTracker Green were then added 10 min prior to imaging at a final concentration of 100 nM.

BODIPY-C_12_ (4,4-difluoro-5,7-dimethyl-4-bora-3a,4a-diaza-*s*-indacene-3-dodecanoic acid): a green color fatty acid analog, BODIPY-NL (4,4-difluoro-1,3,5,7,8-pentamethyl-4-bora-3a,4a-diaza-*s*-indacene): a green color neutral lipid probe, and BODIPY-C_12_ red (4,4-difluoro-5-(2-thienyl)-4-bora-3a,4a-diaza-s-indacene-3-dodecanoic acid): a red color BODIPY fatty acid analog, and LysoTracker Green DND 26 (N-[2-(dimethylamino)ethyl]-3-{2-[(3,5-dimethyl-1H-pyrrol-2-yl-kappaN)methylidene]-2H-pyrrol-5-yl-kappaN}propanamidato)(difluoro)boron), also a BODIPY analog were purchased from ThermoFisher Scientific. Stock solutions were all prepared to 21 µM in DMSO and stored at −20 °C. The dyes were directly added to the medium at the desired nanomolar concentrations (~100 nM was optimal) (see Fig. 2). Unless specified, all yeast samples were imaged ~30 min after the addition of BODIPY dyes.

### Experimental setup and data acquisition

All microscopy experiments were performed with a Nikon Ti-E inverted microscope with a Perfect Focus System. Four imaging lasers (405, 488, 561, and 640 nm, OBIS-CW; Coherent) were combined using dichroic mirrors, aligned, expanded, and focused to the back focal plane of the objective (Nikon-CFI Apo 100× Oil immersion N.A 1.49). The lasers were controlled directly by a computer. A quadband dichroic mirror (zt488/561/640rdc; Chroma) separates the fluorescence emission from the excitation light. All movies (conventional and single molecule) were recorded at a frame rate of 20 Hz on an electron multiplying CCD camera (Ixon89Ultra DU-897U; Andor). The camera was cooled down to −68 °C and the amplifying gain was set to 30. For two-color imaging of red (595 nm) and green fluorescence (525 nm), the fluorescence emission was split by a dichroic longpass beamsplitter (T562lpxr BS; Chroma). The emission in each channel was further filtered by bandpass filters: ET525/50 (Chroma) in the green channel and ET595/50 (Chroma) in the red channel. For simultaneous red and far-red imaging, the emission was split by a dichroic longpass beamsplitter (FF652-Di01; Semrock) and further filtered by bandpass filters: ET610/75 (Chroma) in the red and FF731/137 (Semrock) in the far-red channel.

The 561 nm laser was used to excite single D_II_-states of BODIPY-C_12_, BODIPY-NL (detected in the red emission channel) while a 488 nm laser was used for conventional fluorescence images of the BODIPY-C_12_ and BODIPY-NL monomers (detected in the green emission channel). Shutter sequences were designed to record one conventional image followed by nine single-molecule frames. For mEos2 imaging, the 561 nm excitation laser with the 405 nm activating laser was used. In order to estimate the power density, we measured the power directly at the sample plane and divided by the illumination area. The typical power density for 561 nm excitation of single molecules was 0.8–1 kW cm^−2^, whereas the power density of 488 nm light was 0.035–0.07 W cm^−2^ for conventional fluorescence imaging of BODIPY-C_12_ and BODIPY-NL, and 0.3 W cm^−2^ for conventional imaging of Sec63-2XGFP. The power density for the 405 nm laser was (0.1–1) W cm^−2^. To image single D_II_-states of BODIPY-C_12_ red in the far-red emission channel a 640 nm laser at a power density of ~5 kW cm^−2^ was used, while a 561 nm laser at a power density of ~0.06 W cm^−2^ was used for conventional fluorescence imaging of the monomers in the red emission channel. Again, the repetitive imaging sequence contained one conventional fluorescence frame (561 nm) followed by nine single-molecule frames (640 nm). The super-resolution imaging of BODIPY-C_12_ red (Fig. 4d) was performed using continuous 640 nm laser excitation. Conventional fluorescence images were generated by averaging 100–200 frames. These images came from 488 nm excitation of BODIPY-C_12_, Sec63-2XGFP, BODIPY-NL and 561 nm excitation of BODIPY-C_12_ red. For co-localization quantification of conventional images, the intensities of each pixel from two channels were plotted against each other. The Pearson’s correlation coefficient *ρ* was calculated using the top 60% of the bright pixels in each channel.

The anti-Stokes emission in the shorter wavelength channel was averaged over ~200 frames to produce conventional fluorescence images of the BODIPY monomers. In this case, continuous excitation was used to generate a super-resolution image of D_II_-states in the red shifted emission channel and a conventional (anti-stokes) fluorescence image in the shorter wavelength emission channel (Fig.3b). Similarly, BODIPY-NL and BODIPY-C_12_ red were imaged simultaneously with 488 and 561 nm laser excitation for co-localization under fed and fasted conditions. In order to compare the photon histogram of BODIPY D_II_-states with mEos2, the Faa4-mEos2 was imaged with the same 561 nm power density as BODIPY-C_12_.

### Camera calibration

The EMCCD camera calibration was performed to determine the gain (e/ADU) and the camera offset. The camera pixels were exposed to varying intensity of light with an EMCCD gain of 30. The slope of the mean intensity vs. variance graph accounting for the extra noise factor *F*^2^ was used to calculate the gain^61,62^.The calculated gain of 0.166 e/ADU matched the manufacturers specifications and was used to convert the integrated CCD counts to photons to compare the brightness of D_II_-states with mEos2 at the same 561 nm excitation power.

### Super-resolution and single-molecule tracking data analysis

SMLM analysis was performed using the INSIGHT software (Zhuang lab, Harvard), and cross-validated using the ThunderSTORM^63^ plugin for ImageJ (Fiji). Single-molecule recognition was confirmed by visual perception of fluorescent blinking, and single-molecule identification parameters for 2D Gaussian PSFs were set accordingly (Gaussian height ≥ 50 photons, width (260–650) nm, ROI: 7 × 7 pixels). All single-molecule localizations were rendered as a 2D Gaussian whose width is weighted by the inverse square root of the detected number of photons. All super-resolution images were represented across at least 3000 frames.

Single-molecule traces with a minimum length of 3 frames (20 Hz) were generated by linking consecutive localizations within a 0.48 µm radius. In each frame, an average density of 0.043 localizations per μm^2^ was detected, which ensured a sufficiently low density of localizations to prevent linking different molecules into the same trace. Therefore, the error of accidentally linking two localizations from different molecules within a distance of 0.48 µm is less than 3%. From each trace, the MSD for a particular lag time Δ*t* was calculated by averaging the squared displacements over all time intervals of length Δ*t* with a custom-written Igor-Pro program. The mean diffusion coefficient (*D*) was calculated by linear fitting of the averaged MSD vs. lag-time of >150 traces using <*r*^2^>=4*D*Δ*t* + 2*σ*^2^ for a 2D diffusion equation with localization precision *σ*^64^. The light error-band of the MSD vs. time curve represents the standard error of the mean. Errors of the diffusion coefficients for yeast cells were calculated using standard deviations of the diffusion coefficients calculated from multiple movies (*n* > 5) that contain multiple cells (*n* > 5). For mammalian cells, the error was calculated using the standard deviation of diffusion coefficients calculated from multiple frame ranges (*n* = 6) each containing more than 150 traces.

The size of individual LDs was quantified by averaging the distances of each single-molecule localization from the mean center of all localizations. For the fatty acid distribution in fed and fasted conditions, a radial distribution function was calculated from the localizations to quantify the density of localizations in clusters and their characteristic size. For all radial distribution functions the mean density of localizations was the same to allow a quantitative comparison of the peak height as a measure for clustering.

### SMLM correlation analysis of FA puncta with ER and PM

In order to discriminate whether BODIPY-fatty acid puncta observed in fasting cells are localized to the PM or to the cortical ER (Fig. 5), BODIPY-C_12_ was added to Sec63-mEos2 (ER marker) and PH-mEos2 (PM marker) yeast strains under fasting condition. In order to avoid the cross-talk between mEos2 with D_II_-states, mEos2 localizations were first obtained with 405 nm activation followed by D_II_ imaging without 405 nm. The overlap of D_II_-states localizations during mEos2 imaging was smaller than 20% for the Sec63-mEos2 strain and 30% for PH-mEos2. The radial position of D_II_-states and mEos2 localizations were calculated from the center of each cell for both yeast strains and plotted in a radial distance histogram. The radii of different cells were scaled to be equal in order to combine localizations from different cells in the same radial distance histogram. The separations of the D_II_-states peak in the radial distance histogram relative to Sec63-mEos2 and PH-mEos2 were used to confirm the co-localization of fatty acid puncta and the PM. The cross-correlation function (Supplementary Fig. 5a) was calculated as the relative probability of the pairwise separation of molecules compared to their random distribution inside a cell^65^. This cross-correlation was then used to compare the correlation of D_II_-states with Sec63-mEos2 and PH-mEos2 and to quantify the magnitude of co-localization.

### BODIPY concentration and power optimization

A concentration optimization was performed with BODIPY-C_12_ and BODIPY-NL to find the optimal concentration of BODIPY for SMLM experiments. Each dye was added to separate samples at concentrations of 0 µM (control), 5 nM, 10 nM, 50 nM, 100 nM, 150 nM, 200 nM, and 250 nM. To prevent any differences in the uptake of the dye conjugates, all yeast cells in exponential growth phase were incubated with the dyes at 30 °C for exactly 30 min before imaging and the same 561 nm excitation laser power was used for SMLM experiments at all concentrations (each data set contained >10 cells and >3000 frames). The resulting single-molecule detections per cell per second were used to determine optimal BODIPY concentrations. The standard deviation of the mean detections per cell per frame was calculated by analyzing five separate substacks of the movie each consisting of an equal number of frames. To find the optimal laser excitation power for super-resolution imaging of D_II_-BODIPY-C_12_, an optimal BODIPY-C_12_ concentration of 100 nM was used. To minimize potential phototoxic effects, the lowest 561 nm laser power was used first and the power was then incrementally increased. The same cells were imaged at each laser power for a minimum of 3000 frames at 20 Hz and each dataset was taken as quickly as possible to avoid any prominent changes in cellular conditions over time. All data sets were analyzed using the ThunderSTORM plugin for ImageJ (Fiji), and for each power the localization precision was calculated using the formula by Thompson et al.

### Reporting summary

Further information on research design is available in the Nature Research Reporting Summary linked to this article.

## Supplementary information

Supplementary Information

Description of Additional Supplementary Files

Supplementary Movie 1

Supplementary Movie 2

Reporting Summary

## Data Availability

All raw SMLM movie files, yeast strains and plasmids from this study are available from the corresponding author upon reasonable request.

## Code availability

Conventional and super-resolution microscopy data were collected with HAL4000 and analyzed with Insight3, which is available from Xiauwei Zhuang’s lab (Harvard) at: http://zhuang.harvard.edu. Conventional fluorescence microscopy data were analyzed with the freely available Fiji https://imagej.net. Plots and statistical analysis were performed with Matlab R2018b and IgorPro 6.37 procedures, which will be made available by the corresponding author upon reasonable request.
