# Supplementary material for: Single-molecule localization microscopy and tracking with red-shifted states of conventional BODIPY conjugates in living cells
Source: Nat Commun. 2019 Jul 30;10:3400. doi: 10.1038/s41467-019-11384-6 (PMC6667493; doi:10.1038/s41467-019-11384-6)
Supplement: Supplementary file 1 — Supplementary Information [file 41467_2019_11384_MOESM1_ESM.pdf]

Supplementary Information

Single-molecule localization microscopy and tracking with red-shifted states of conventional BODIPY conjugates in living cells

Santosh Adhikari[1], Joe Moscatelli[1], Elizabeth M. Smith[1], Chiranjib Banerjee[1] and Elias M. Puchner[1#]

[1]School of Physics and Astronomy

University of Minnesota, Twin Cities

Physics and Nanotechnology (PAN)

115 Union Street SE, Minneapolis, MN 55455

# corresponding author, e-mail: epuchner@umn.edu

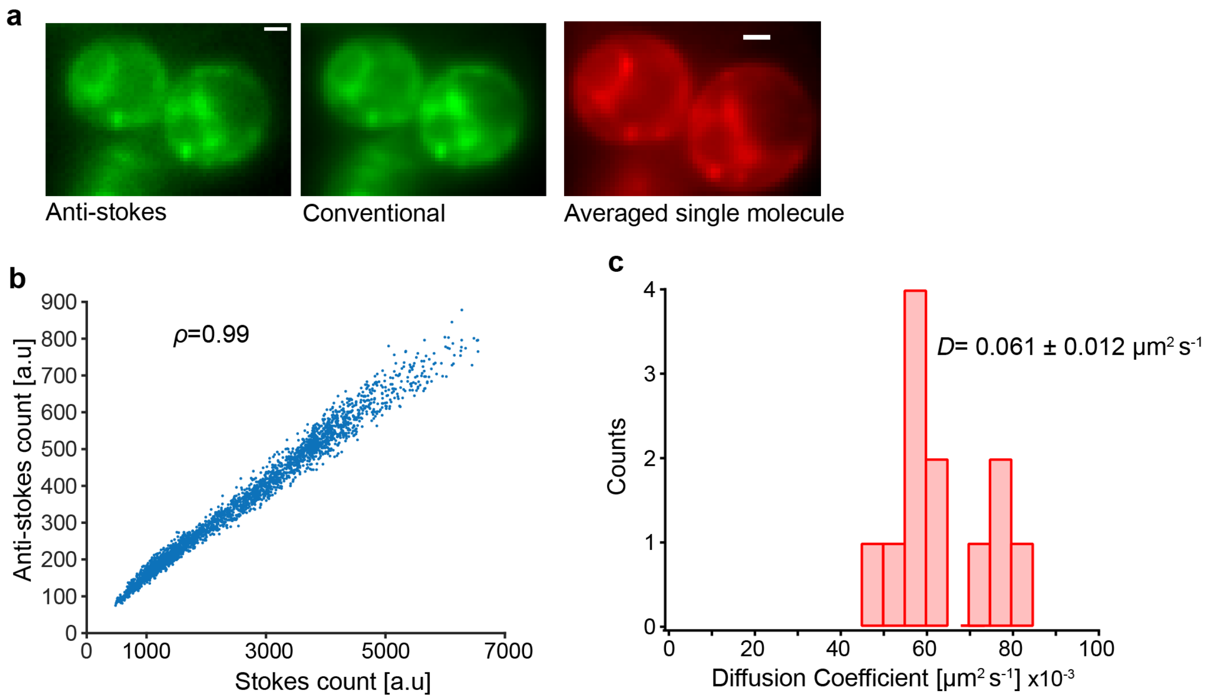

Suppl. Fig.1| The co-localization of anti-stokes, conventional bulk fluorescence and single-molecule fluorescence images of BODIPY-C_{12} and the distribution of diffusion coefficients of fatty acid analogs using single-molecule tracking of $D_{||}$ states of BODIPY-C_{12}. a (left) Anti-stokes fluorescence image of BODIPY-C_{12} (ex. 561 nm, em. 525 nm) averaged over 200 frames. (center) Conventional fluorescence image (ex. 488 nm, em. 525 nm) averaged over 200 frames. (right) Single-molecule fluorescence (ex. 561 nm, em. 595 nm) image averaged over 800 frames. **b** The correlation of conventional and anti-stokes fluorescence images shows the same source of the signal (Person's ρ=0.99*,* n= 4160 points, 3 cells). **c** The distribution of diffusion coefficients has a mean of D= 0.061 ± 0.012 μm^2/s. Each of the 12 diffusion coefficients was calculated from a different movie containing multiple cells (n>3). For each diffusion coefficient, multiple traces (n>150), each lasting at least three data acquisition frames, were used to calculate the MSD vs. time curve, which was fitted with a straight line. The error represents the standard deviation of diffusion coefficients.

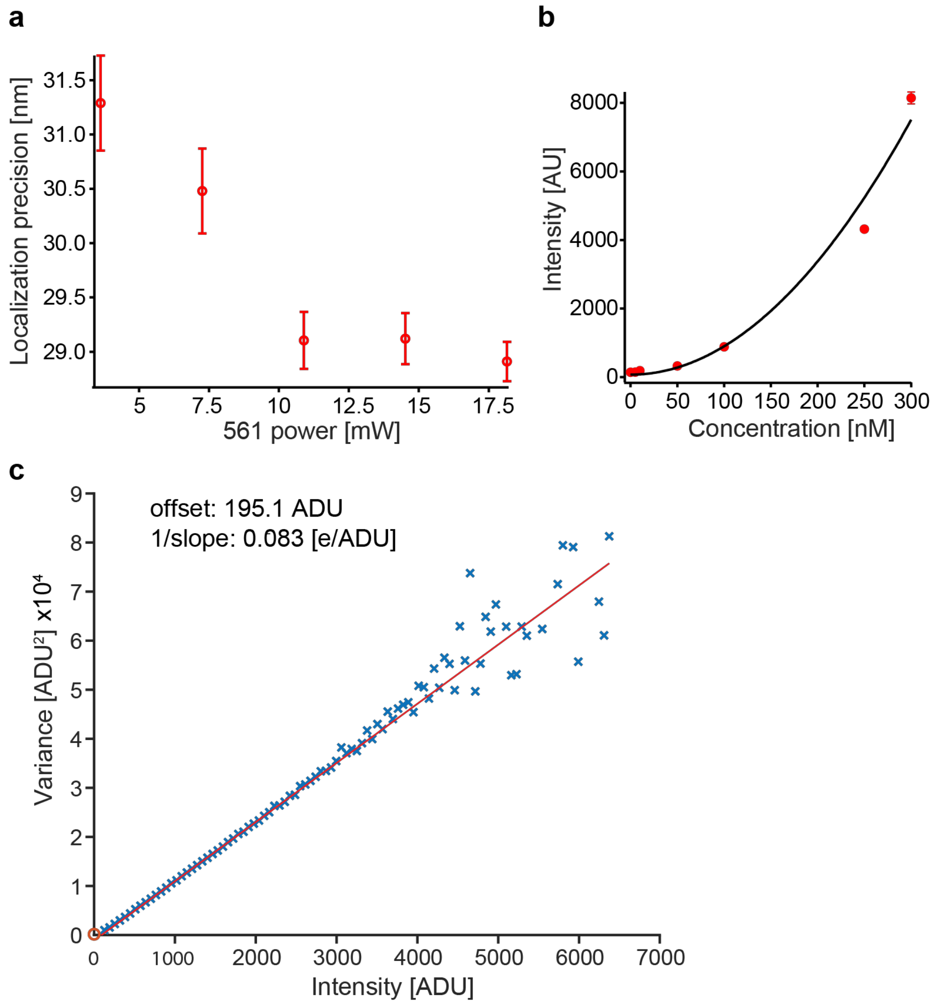

Suppl. Fig. 2| Characterization and optimization of BODIPY-C_{12} for single-molecule imaging. a Mean localization precision for different 561 nm excitation powers. Each data point contains more than 400 single-molecule localizations. Error bars represent the standard error of the mean localization precision. **b** The mean intensity of the fluorescent background of $D_{||}$-BODIPY-C_{12} (ex. 561 nm) averaged over 400 pixels and 500 frames. Error bars represent standard deviation in time. The quadratic dependence of background fluorescence on the concentration suggests dimerization mechanism for forming $D_{||}$-states. **c** Mean intensity vs variance of camera pixels exposed to various intensities shows a linear behavior with an inverse slope of 0.083 e/ADU. The extra noise Factor (F^2=2) for EMCCD gain of 30 gives a conversion factor of 0.166 e/ADU which is used for converting integrated counts to photons and is consistent with the manufacturers specification.

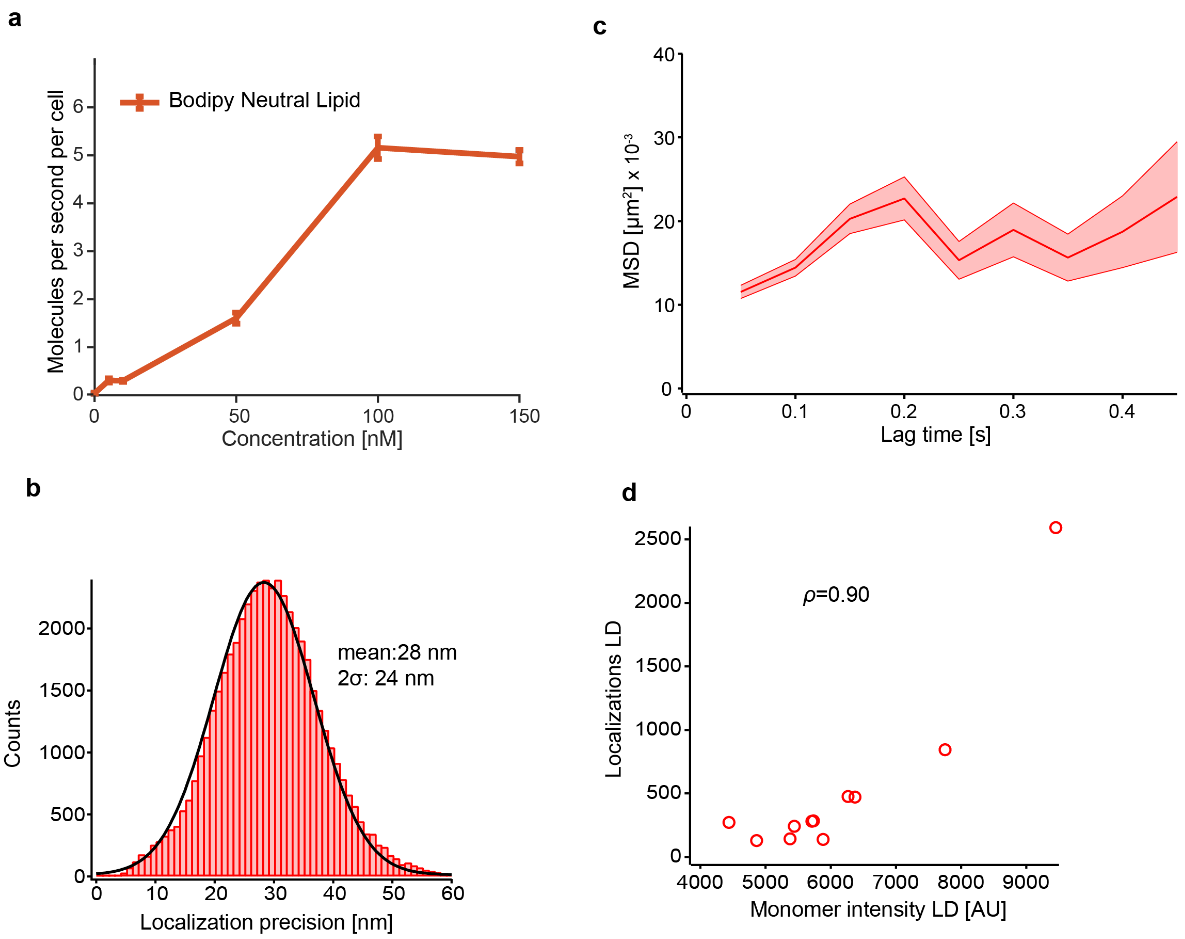

Suppl. Fig. 3| Characterization of $D_{||}$-states of BODIPY-NL and quantification of LDs. a The rate of single-molecule detections is influenced by the BODIPY-NL concentration (n>10 cells, 5000 frames each). **b** The theoretical single-molecule localization precision calculated with the formula by Thompson et al. has a mean of 28 nm. **c** The single-molecule tracking of BODIPY-NL is used to compute MSD vs. time plot. The nature of the plot shows a confined diffusion of BODIPY-NL molecules inside the LDs (n=208 traces). Error band represents the standard error of the mean displacement. **d** The quantification of the monomer intensity and the number of localizations for individual LDs of different size shows a correlation indicating that the dimer population can be used for quantification.

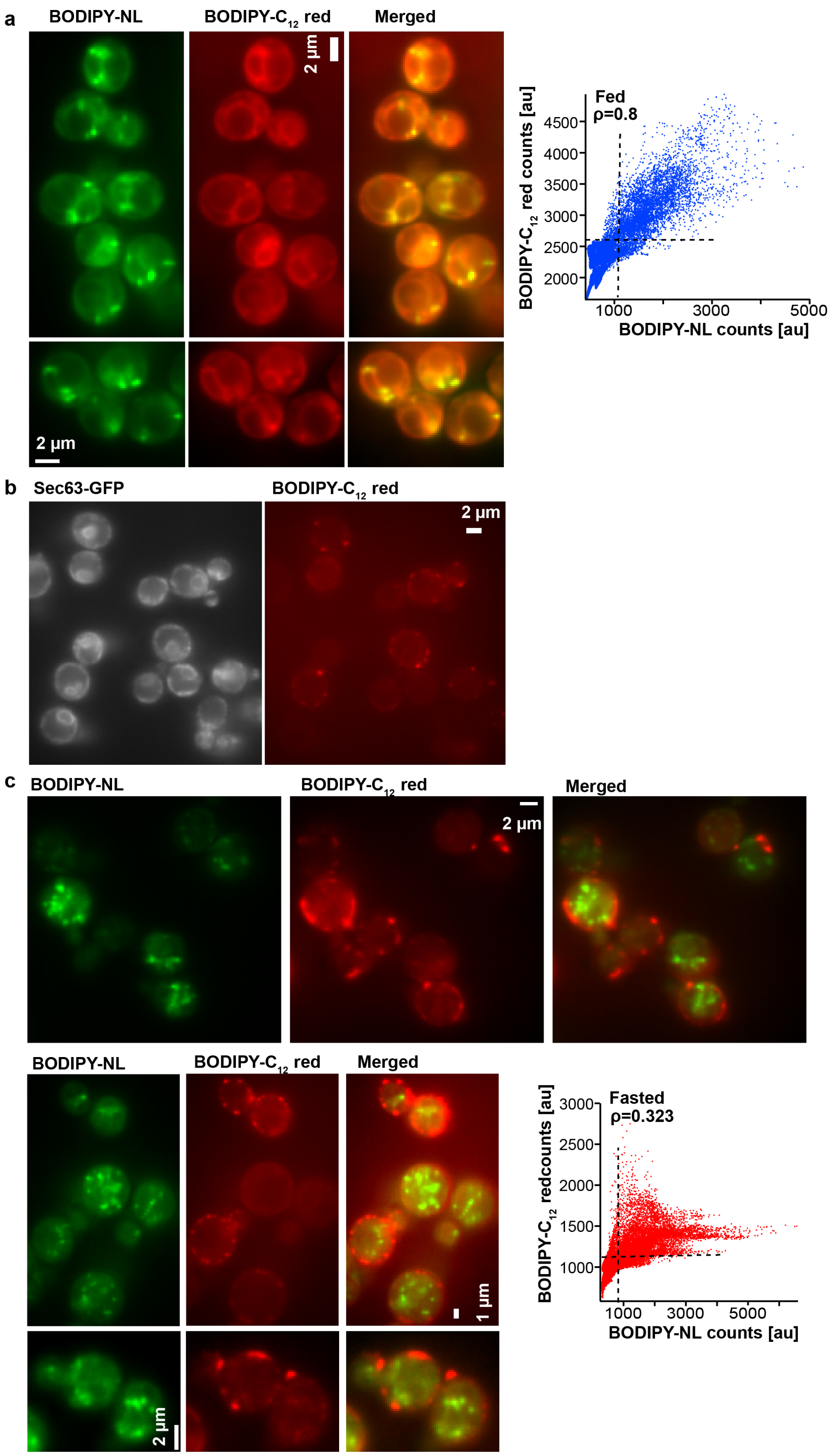
a
BODIPY-NL
BODIPY-C12 red
Merged
2 µm
2 µm
Fed
ρ=0.8
BODIPY-C12 red counts [au]
BODIPY-NL counts [au]
b
Sec63-GFP
BODIPY-C12 red
2 µm
c
BODIPY-NL
BODIPY-C12 red
Merged
2 µm
BODIPY-NL
BODIPY-C12 red
Merged
1 µm
2 µm
Fasted
ρ=0.323
BODIPY-C12 redcounts [au]
BODIPY-NL counts [au]

Suppl. Fig. 4| Differential localization of fatty acid analogs under fed and fasted condition. a Conventional fluorescence images (average of 200 frames) of BODIPY-NL and BODIPY-C_{12} red under fed condition. The distinct spots from BODIPY-NL co-localize (Pearson's ρ=0.8, 10 cells) with clusters of BODIPY-C_{12} red along the ER indicating the incorporation of fatty acid analogs in LDs. **b** Conventional fluorescence images (average of 50 frames) of BODIPY-C_{12} red and Sec63-GFP under fasted condition. The BODIPY-C_{12} red shows distinct puncta around cell periphery. **c** The conventional fluorescence images of BODIPY-C_{12} red and BODIPY-NL under fasted condition. The neutral lipid signal from LDs shows no co-localization (Pearson's ρ=0.3,10 cells) with distinct puncta from BODIPY-C_{12} red around the cell periphery.

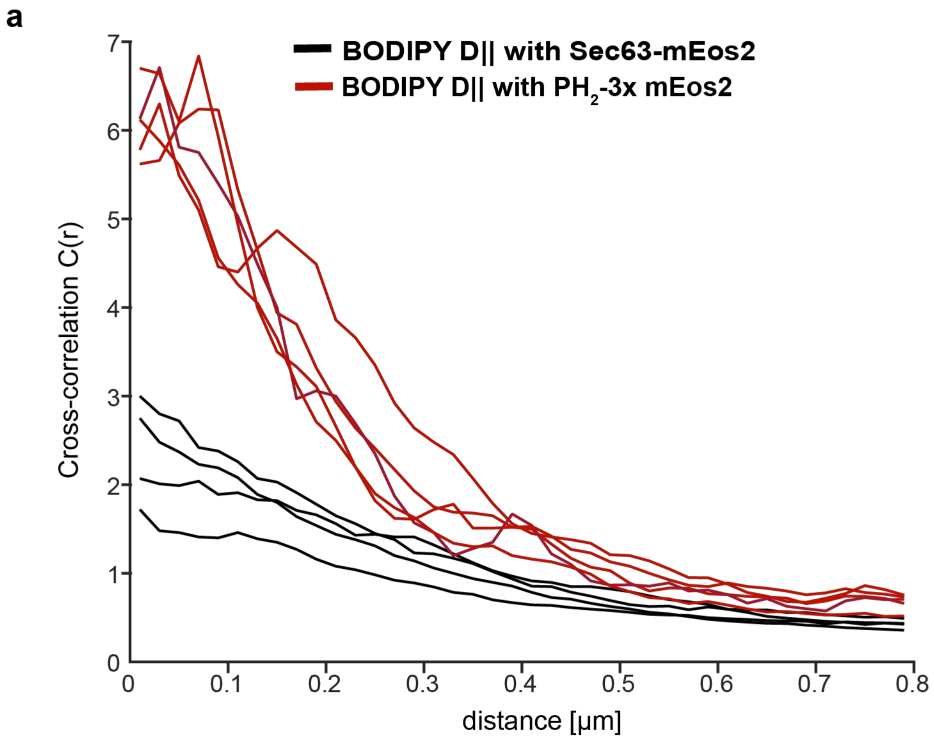

Suppl. Fig. 5| $D_{||}$ state signal from BODIPY-C_{12} red shows higher cross-correlation with PH_2-3x mEos2 compared to Sec63-mEos2. **a** The cross-correlation of $D_{||}$-BODIPY-C_{12} red with Sec63-mEos2 (black) and with PH_2-3x mEos2 (red). The correlation with PH_2-3x mEos2 is stronger (~2 times) compared to Sec63-mEos2 at short distances (~20-80 nm). The different curves represent cross-correlations from different cells.

a

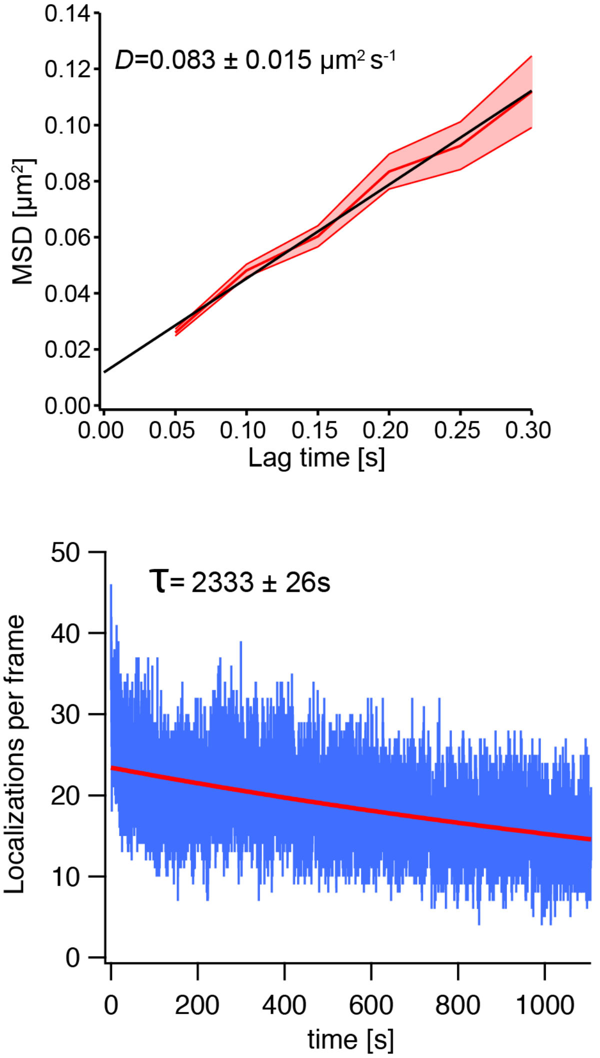

Suppl. Fig. 6| Detection rate and tracking of $D_{||}$-BODIPY-C_{12} states in U2OS cells. a The averaged MSD vs. time plot of single-molecule traces shows free diffusion of BODIPY-C_{12} with a mean diffusion coefficient of D=0.083 ± 0.015 $\mu m^2/s$. The diffusion coefficient was calculated from a linear fit (black line). The error band represents the standard error of the mean after averaging over 200 traces each lasting at least three acquisition frames (Error in diffusion coefficient: standard deviation from 6 different frame ranges each with minimum 150 traces from 1 cell). **b** The detection rate of single-molecules vs. data acquisition time shows that cells can be imaged for many minutes without a significant decrease in localizations.
