# Supplementary material for: Single-molecule localization microscopy and tracking with red-shifted states of conventional BODIPY conjugates in living cells
Source: Nat Commun. 2019 Jul 30;10:3400. doi: 10.1038/s41467-019-11384-6 (PMC6667493; doi:10.1038/s41467-019-11384-6)
Supplement: Supplementary file 2 — Description of Additional Supplementary Files [file 41467_2019_11384_MOESM2_ESM.pdf]

Description of Additional Supplementary Files

File Name: Supplementary Movie 1
Description: Raw movie file of imaging single DII-BODIPY-C_{12} states.

File Name: Supplementary Movie 2
Description: Conventional fluorescence movie of BODIPY-NL excited at 488 nm under fasting conditions. LDs are either tethered to the vacuole or exhibit fast diffusion inside the vacuole.
