# Supplementary material for: Single-molecule localization microscopy and tracking with red-shifted states of conventional BODIPY conjugates in living cells
Source: Nat Commun. 2019 Jul 30;10:3400. doi: 10.1038/s41467-019-11384-6 (PMC6667493; doi:10.1038/s41467-019-11384-6)
Supplement: Supplementary file 5 — Reporting Summary [file 41467_2019_11384_MOESM5_ESM.pdf]

nature research

Corresponding author(s): Elias M. Puchner

Last updated by author(s): Jul 1, 2019

Reporting Summary

Nature Research wishes to improve the reproducibility of the work that we publish. This form provides structure for consistency and transparency in reporting. For further information on Nature Research policies, see Authors & Referees and the Editorial Policy Checklist.

Statistics

For all statistical analyses, confirm that the following items are present in the figure legend, table legend, main text, or Methods section.

n/a	Confirmed	
☐	☒	The exact sample size (*n*) for each experimental group/condition, given as a discrete number and unit of measurement
☐	☒	A statement on whether measurements were taken from distinct samples or whether the same sample was measured repeatedly
☒	☐	The statistical test(s) used AND whether they are one- or two-sided<br>*Only common tests should be described solely by name; describe more complex techniques in the Methods section.*
☒	☐	A description of all covariates tested
☒	☐	A description of any assumptions or corrections, such as tests of normality and adjustment for multiple comparisons
☐	☒	A full description of the statistical parameters including central tendency (e.g. means) or other basic estimates (e.g. regression coefficient) AND variation (e.g. standard deviation) or associated estimates of uncertainty (e.g. confidence intervals)
☒	☐	For null hypothesis testing, the test statistic (e.g. *F*, *t*, *r*) with confidence intervals, effect sizes, degrees of freedom and *P* value noted<br>*Give P values as exact values whenever suitable.*
☒	☐	For Bayesian analysis, information on the choice of priors and Markov chain Monte Carlo settings
☒	☐	For hierarchical and complex designs, identification of the appropriate level for tests and full reporting of outcomes
☐	☒	Estimates of effect sizes (e.g. Cohen's *d*, Pearson's *r*), indicating how they were calculated

Our web collection on statistics for biologists contains articles on many of the points above.

Software and code

Policy information about availability of computer code

Data collection	Conventional and Super-resolution microscopy data was collected with HAL4000, which is available from Xiauwei Zhuang's lab (Harvard) http://zhuang.harvard.edu
Data analysis	Super-resolution data was analyzed with Insight3, the super-resolution microscopy software available from Xiauwei Zhuang's lab (Harvard) http://zhuang.harvard.edu. Conventional fluorescence microscopy data was analyzed with the freely available Fiji https://imagej.net. Plots and statistical analysis was performed with Matlab R2018b and IgorPro 6.37.

For manuscripts utilizing custom algorithms or software that are central to the research but not yet described in published literature, software must be made available to editors/reviewers. We strongly encourage code deposition in a community repository (e.g. GitHub). See the Nature Research guidelines for submitting code & software for further information.

Data

Policy information about availability of data

All manuscripts must include a data availability statement. This statement should provide the following information, where applicable:

- Accession codes, unique identifiers, or web links for publicly available datasets
- A list of figures that have associated raw data
- A description of any restrictions on data availability

One example of a raw SMLM movie file is available as a supplementary file, all other raw (SMLM) movie files are available from the corresponding author upon reasonable request.

Field-specific reporting

Please select the one below that is the best fit for your research. If you are not sure, read the appropriate sections before making your selection.

☒ Life sciences ☐ Behavioural & social sciences ☐ Ecological, evolutionary & environmental sciences

For a reference copy of the document with all sections, see nature.com/documents/nr-reporting-summary-flat.pdf

Life sciences study design

All studies must disclose on these points even when the disclosure is negative.

Sample size	All sample sizes are described in the figure captions or Materials and Methods section. In most cases the different samples were different cells, in which case the number of cells was counted by visual inspection of the image files. In cases where the samples consist of multiple localizations or traces, the number of localizations or traces is noted.
Data exclusions	The first ~200 frames of SMLM movies were not analyzed to allow for bleaching of background fluorescence as it is commonly done in PALM/STORM experiments. In rare cases few yeast cells (or lipid droplets) in the field of view exhibited movement during the data acquisition. These moving cells (or lipid droplets) were excluded from data analysis to avoid blurring artifacts.
Replication	Replication of the SMLM capability of conventional BODIPY conjugates was achieved across three different BODIPY conjugates, two colors and two different cell types. Overall we recorded 60 different SMLM movies from 10 independent experiments performed on different days. Biological findings on the differential localization of LDs and fatty acid analogs were replicated in the following way: 20 movies of BODIPY-C12, 10 movies of BODIPY-NL and 10 movies of BODIPY-C12 in fed cells; 10 movies of BODIPY-C12 red and 5 movies of BODIPY-NL in fasted cells. All data sets and observations replicated the results and no data set contradicted any reported finding.
Randomization	n/a
Blinding	n/a

Reporting for specific materials, systems and methods

We require information from authors about some types of materials, experimental systems and methods used in many studies. Here, indicate whether each material, system or method listed is relevant to your study. If you are not sure if a list item applies to your research, read the appropriate section before selecting a response.

Materials & experimental systems

n/a	Involved in the study
☒	☐ Antibodies
☐	☒ Eukaryotic cell lines
☒	☐ Palaeontology
☒	☐ Animals and other organisms
☒	☐ Human research participants
☒	☐ Clinical data

Methods

n/a	Involved in the study
☒	☐ ChIP-seq
☒	☐ Flow cytometry
☒	☐ MRI-based neuroimaging

Eukaryotic cell lines

Policy information about cell lines

Cell line source(s)	The parental s.cerevisiae yeast cell line W303_ADE+ was purchased and authenticated from Horizon-Dharmacon. The U2OS cell line was purchased and authenticated by ATCC (kind gift from Jochen Mueller's lab).
Authentication	The cell lines were authenticated by the companies where they were purchased (yeast: Horizon-Dharmacon; U2OS:ATCC)
Mycoplasma contamination	All cells tested negative for mycoplasma contamination as evident from Hoechst staining and healthy morphology of cells seen in the microscope.
Commonly misidentified lines (See ICLAC register)	No commonly misidentified cell lines were used in this study
